# SPAS-1 expression in neurons and vulva during L4 stage​

**DOI:** 10.17912/W2P94X

**Published:** 2017-07-31

**Authors:** George Brown, Rachid El Bejjani

**Affiliations:** 1 Department of Biology, Davidson College, Box 7118, Davidson, NC 28035, USA.

**Figure 1.**
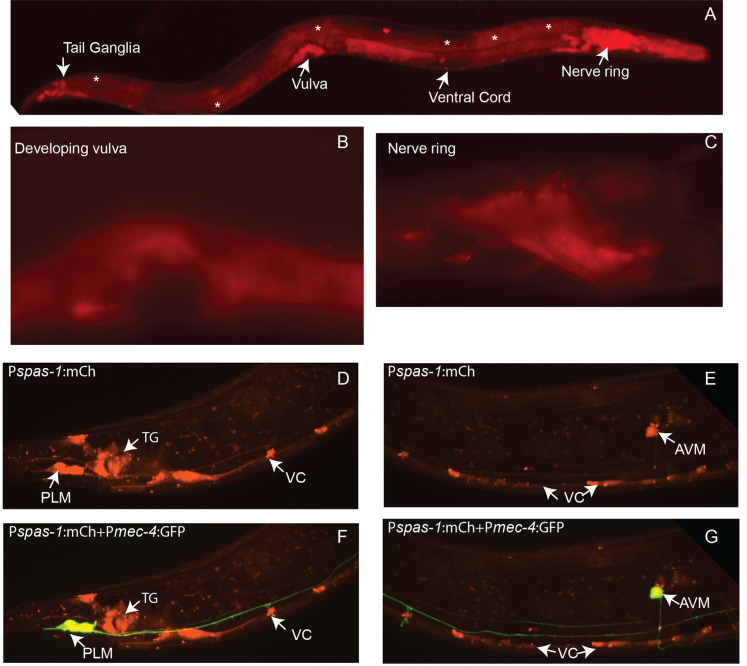


## Description

*spas-1* is the worm homolog of the human spastic paraplegia gene, spastin. Previous work in *C. elegans* has shown that SPAS-1 disassembles microtubules in an ATP-dependent manner (Matsushita-Ishiodori *et al,* 2007). Another study shows that SPAS-1 plays a role in synapse remodeling in motor neurons (Kurup *et al,* 2015). Here we describe the expression of SPAS-1 in L4 hermaphrodite larvae using the transcriptional reporter, *axrEx8*[P*spas-1*:mCh]: A) Low magnification (10X objective) image of an entire L4 larva. mCherry expression is driven by the *spas-1* promoter (sequence provided). Expression is observed in the nerve ring, ventral cord, vulva, and tail ganglia. Expression is also detected in the intestine (asterisks). B) High magnification (100X objective) image of SPAS-1 expression in the developing vulva of an L4 hermaphrodite. C) High magnification (100X objective) image of nerve ring expression. D-F) SPAS-1 expression in the tail ganglia (TG), ventral cord (VC), and mechanosensory neurons (PLM and AVM shown here). Mechanosensory neuron expression is confirmed by overlaying GFP and mCherry fluorescence in *axrEx8* [P*spas-1*:mCh]; *zdIs5* [P*mec-4*:GFP + *lin-15* (+)] animals. Arrows point at the following mechanosensory neurons: PLM=Posterior Lateral Microtubule cell, AVM=Anterior Ventral Microtubule cell.

Our work shows that SPAS-1 is expressed in several neuronal types as well as in the intestine and developing vulva in L4 larvae. These findings complement expression data by Matsushita-Ishiodori who focused on SPAS-1 expression in embryos.

## Reagents

Transgenes: *axrEx8*; *zdIs5*.

*axrEx8* [P*spas-1*:mCh] transcriptional fusion construction details: 480bp upstream of the *spas-1* ATG cloned into Pdonr4-1 gateway vector. Expression clone built by LR multisite recombination to drive mCherry expression. Strain REB22, injection concentration 20ng/ul.

*spas-1* promoter sequence: provided by the authors and included in the transgene details.
